# The distinct role of CD73 in the progression of pancreatic cancer

**DOI:** 10.1007/s00109-018-01742-0

**Published:** 2019-03-29

**Authors:** Liangjing Zhou, Shengnan Jia, Yan Chen, Weiming Wang, Zhengrong Wu, Weihua Yu, Mingjie Zhang, Guoping Ding, Liping Cao

**Affiliations:** Department of General Surgery, Sir Run Run Shaw Hospital, School of Medicine, Zhejiang University, No. 3, Qingchun Road, Hangzhou, 310000 China

**Keywords:** Pancreatic cancer, Progression, CD73, TNFR2, miR-30a-5p

## Abstract

**Abstract:**

Recent studies have shown that the non-enzymatic function of CD73 plays a key role in tumor progression, but this function of CD73 in pancreatic cancer cells has not been studied. Furthermore, little is known about the mechanism involved in CD73 regulation in tumors. Here, we found that CD73 expression was upregulated in pancreatic ductal adenocarcinoma (PDAC) and that its expression correlated with poor prognosis. CD73 knockdown inhibited cell growth and induced G1 phase arrest via the AKT/ERK/cyclin D signaling pathway. We also found that tumor necrosis factor receptor (TNFR) 2 was involved in CD73-induced AKT and ERK signaling pathway activation in PDAC. Further, miR-30a-5p overexpression significantly increased the cytotoxic effect of gemcitabine in pancreatic cancer by directly targeting CD73 messenger RNA (mRNA), suggesting that regulation of the miR-30a-5p/CD73 axis may play an important role in the development of gemcitabine resistance in pancreatic cancer. In summary, this regulatory network of CD73 appears to represent a new molecular mechanism underlying PDAC progression, and the mechanistic interaction between miR-30a-5p, CD73, and TNFR2 may provide new insights into therapeutic strategies for pancreatic cancer.

**Key messages:**

- CD73 was upregulated in PDAC and correlated with poor prognosis.
- CD73 knockdown inhibited cell growth and induced G1 phase arrest.
- TNFR2 was involved in CD73-induced AKT and ERK signaling pathway.
- miR-30a-5p targeted CD73 and increased the sensitivity to gemcitabine.

**Electronic supplementary material:**

The online version of this article (10.1007/s00109-018-01742-0) contains supplementary material, which is available to authorized users.

## Introduction

Pancreatic cancer is the fourth leading cause of cancer-related death in the USA, with a 5-year survival rate of only 3% to 6%. Up to 85.3% of deaths occurred in newly diagnosed cases, ranking first in all common malignancies [1]. Most patients present with metastatic disease at the time of diagnosis, and only 20% are eligible for surgery [2, 3]. Thus, further exploration of new biomarkers and elucidation of the underlying mechanism of pancreatic cancer are urgently needed.

CD73 is a cell surface protein encoded by the NT5E gene that plays a crucial role in adenosinergic signaling. CD73 has both enzymatic and non-enzymatic functions in cells [4, 5]. Notably, increasing data have shown that CD73 is also a key regulator involved in the progression of cancer [6, 7]. Gao et al. [8] found that CD73 promotes the proliferation and migration of human cervical cancer cells independent of its enzymatic activity. However, the non-enzymatic function of CD73 has not been well studied. Previous studies demonstrated that CD73 is overexpressed in many types of cancer cell lines and promotes tumor progression [9–18]. However, the precise role of CD73 in pancreatic cancer remains unclear and the mechanism of CD73 in tumor progression requires further exploration.

In the present study, we examined the expression of CD73 in pancreatic cancer and its underlying mechanisms. Our findings may provide new insights into developing novel therapeutic strategies for pancreatic ductal adenocarcinoma (PDAC).

## Materials and methods

### Cell culture

Human pancreatic cancer cell line, including Panc-1, AsPC-1, SW1990, BxPC-3, MIAPaCa-2, and CFPAC-1, and normal pancreatic ductal epithelial cells (HPDE6-C7) were all obtained from Chinese Academy of Sciences (Shanghai, China) and were cytogenetically tested and authenticated during the past 3 months. PANC-1, SW1990, MIAPaCa-2, and HPDE6-C7 cell lines were cultured in Dulbecco’s modified Eagle’s medium (DMEM; Invitrogen) with 10% fetal bovine serum (FBS). BxPC-3 and AsPC-1 cell lines were cultured in RPMI 1640 medium containing 10% FBS. CFPAC-1 cell line was cultured in IMDM medium with 10% FBS.

### Human tissue specimens and immunocytochemistry staining

Pancreatic ductal adenocarcinoma and adjacent normal pancreatic tissue were obtained from pancreatic cancer patients between 2012 and 2015. The research protocol was reviewed and approved by the Research Ethics Committee of Sir Run Run Shaw Hospital, School of Medicine, Zhejiang University. All participants or their guardians gave written consent of their tissue samples and medical information to be used for scientific research. Immunohistochemical staining was carried out using the biotin-streptavidin method. The primary antibodies used were CD73 (1:100, sc-32299; Santa Cruz), tumor necrosis factor receptor (TNFR) 2 (1:100, ab109322; Abcam), and Ki-67 (1:250, ab16667; Abcam).

The scoring procedure was carried out twice by two independent pathologists without any knowledge of the clinical data. The staining of CD73 was evaluated under a light microscope at a magnification of × 400 according to the intensity of staining and the percentage of stained cells. Five random fields were examined for each tumor specimen. The staining density was scored as follows: 0 indicates negative, no, or less than 5% of positive cells; 1 indicates 5–25%of positive cells; 2 indicates 26–50% of positive cells; and 3 indicates more than 50% of positive cells. The dominant staining intensity was scored as follows: 0 indicates negative, 1 indicates weak, 2 indicates intermediate, and 3 indicates strong staining intensity. Expression was calculated by adding density score (0–3) and intensity score (0–3) before categorizing into low and high expression levels. High expression was defined as score ≥ 3.

### Transient transfection

CD73 small interfering RNA (siRNA) (siG000004907A), TNFR2 siRNA (stQ0003462-1), miR-30a-5p mimics (miR10000087-1-5), inhibitor (miR20000087-1-5), and their matched negative control were synthesized by Guangzhou RiboBio Co., Ltd. (Guangzhou, China). Recombinant plasmids overexpressing CD73 and matched negative control were constructed by Shanghai Genechem Co., Ltd. (GOCP2301062043). Transfection was performed using Lipofectamine 3000 following the manufacturer’s protocol (Invitrogen, California, USA).

### Cell population doubling time

Cells were seeded in six-well plates and allowed to grow. The cells were harvested by trypsinization and counted every day for 5 days. Viable cells were counted using a hemocytometer. All experiments were performed in the logarithmic phase of cell growth. The cell population doubling time (*T*_d_) was calculated during the exponential growth phase (48–96 h) using the following formula: *T*_d_ = 0:693*t* / log(*N*_*t*_/*N*_0_).

### Flow cytometric assessment of apoptosis

Cells were collected by trypsinization and washed three times using PBS, and then cells were treated with annexin V-PE and 7AAD for 30 min according to the instruction manual elsewhere. Samples were acquired on a BD FACSAria III Cell Sorting System (Becton Dickinson, New York, USA) before analysis using the BD FACSDiva software 6.1.3 (Becton Dickinson).

### Cell cycle analysis and synchronization

Cells were harvested and washed with PBS, fixed with 75% ethanol overnight at 4 °C, and then incubated with RNase at 37 °C for 30 min. The nuclei of the cells were stained with propidium iodide (PI) for 30 min and analyzed with a fluorescence-activated cell sorting caliber system. The results were presented as the percentages of cells in each phase.

Cell synchronization was performed as described previously [19], and protein was prepared for each hour post-release from the arrest.

### RNA extraction and qRT-PCR

Total RNA of cells was isolated and purified by miRNeasy Mini Kit (Qiagen, Maryland, USA), following the manufacturer’s instructions. RT was performed using PrimeScript RT Reagent Kit (Takara, Otsu, Japan) following the manufacturer’s instructions. The polymerase chain reaction (PCR) primers of miR-30a-5p and U6 used were purchased from Guangzhou RiboBio Co., Ltd. (MQP-0101 and MQP-0202; Guangzhou, China); the CD73, TNF-α, and GAPDH primers were designed as follows: CD73 forward 5′-GCATTCCTGAAGATCCAAGC-3′ and reverse 5′-GATTGAGAGGAGCCATCCAG-3′, TNF-α forward 5′-AGCTGGTGGTGCCATCAGAGG-3′ and reverse 5′-TGGTAGGAGACGGCGATGCG-3′, and GAPDH forward 5′-GCACCGTCAAGGCTGAGAAC-3′ and reverse 5′-TGGTGAAGACGCCAGTGGA-3′.

### Detection of TNF-α levels in supernatant via ELISA

Supernatant was investigated for levels of TNF-α protein using commercial TNF-α ELISAs (Hengyuan Biotechnology Co., Ltd., Shanghai, China) according to the manufacturer’s instructions.

### Western blot

Cells were harvested, and total protein lysates were resolved using 10% sodium dodecyl sulfate–polyacrylamide gels, electroblotted onto a polyvinylidene fluoride membrane, blocked by 5% skim milk, and probed with primary antibodies listed in Table S1. The membrane was then incubated with the secondary antibody and visualized by enhanced chemiluminescence using Kodak X-Omat LS film (Eastman Kodak, Rochester, USA).

### Luciferase assay

Cells were co-transfected with pLMP vectors containing CD73 3′-untranslated region (UTR) and miR-30a-5p mimics or inhibitors. Cells were harvested and subjected to lysis 48 h after transfection. Renilla luciferase activity was used for normalization, and firefly luciferase activity was detected with a dual-luciferase reporter assay kit according to the manufacturer’s protocol.

### Animal experiments and bioluminescence imaging

PDAC-bearing nude mice with subcutaneous passage of PANC-1-luciferin were used. When the tumor size reached approximately 5 mm in length, the mice were divided into two groups (four mice per group) randomly. Stabilized microRNAs (miRNAs) (miR-30a-5p agomir and negative control agomir) were purchased from RiboBio (miR40000087-1-10; Guangzhou, China). miR-30a-5p agomir (5 nM) or control oligo mixture was injected into the xenografts in a multi-site injection manner every 3 days for 2 weeks. The tumor volume was measured with a caliper every 3 days, using the following formula: volume = length × width^2^/2. At the end of the 30-day observation period, the mice bearing tumor xenografts were examined by bioluminescence imaging and then sacrificed and the tumor tissues were removed for formalin fixation.

The animals anesthetized by isoflurane were intraperitoneally injected with d-luciferin (Biotium, USA) in a concentration of 150 mg/kg and, 20 min later, were subjected to the in vivo bioluminescence imaging using the system of photobiology.

### Fluorescence in situ hybridization detection of miR-30a-5p in tumor sections

FISH was performed based on a previously described protocol [20]. For the detection of expression levels of miR-30a-5p in tumor sections, the following LNA oligo sequences were used: LNA-miR-30a-5p 5′-UGUAAACAUCCUCGACUGGAAG-3′, LNA-U6 5′-TTTGCGTGTCATCCTTGCG-3′, and scramble 5′-GTGTAACACGTCTATACGCCCA-3′, which were constructed by GenePharma Corporation (Shanghai, China).

### In vitro cytotoxicity assay

PANC-1 and CFPAC-1 cells were plated in a 96-well plate and treated after 72 h with gemcitabine (Eli Lilly Corporation, Indianapolis, USA). The absorbance at 450 nm was measured using a microplate reader. Drug sensitivity curves and IC_50_ values were calculated using GraphPad Prism 5.0 software (GraphPad Software, San Diego, CA).

### Statistical analysis

All data were presented as mean ± standard deviation (SD) and analyzed using Student’s *t* test. A *p* value < 0.05 was considered as statistically significance. All data were processed using SPSS (version 19.0) and GraphPad Prism 5.0 software program.

## Results

### CD73 is overexpressed in PDAC tissues and cell lines

**Fig. 1:**
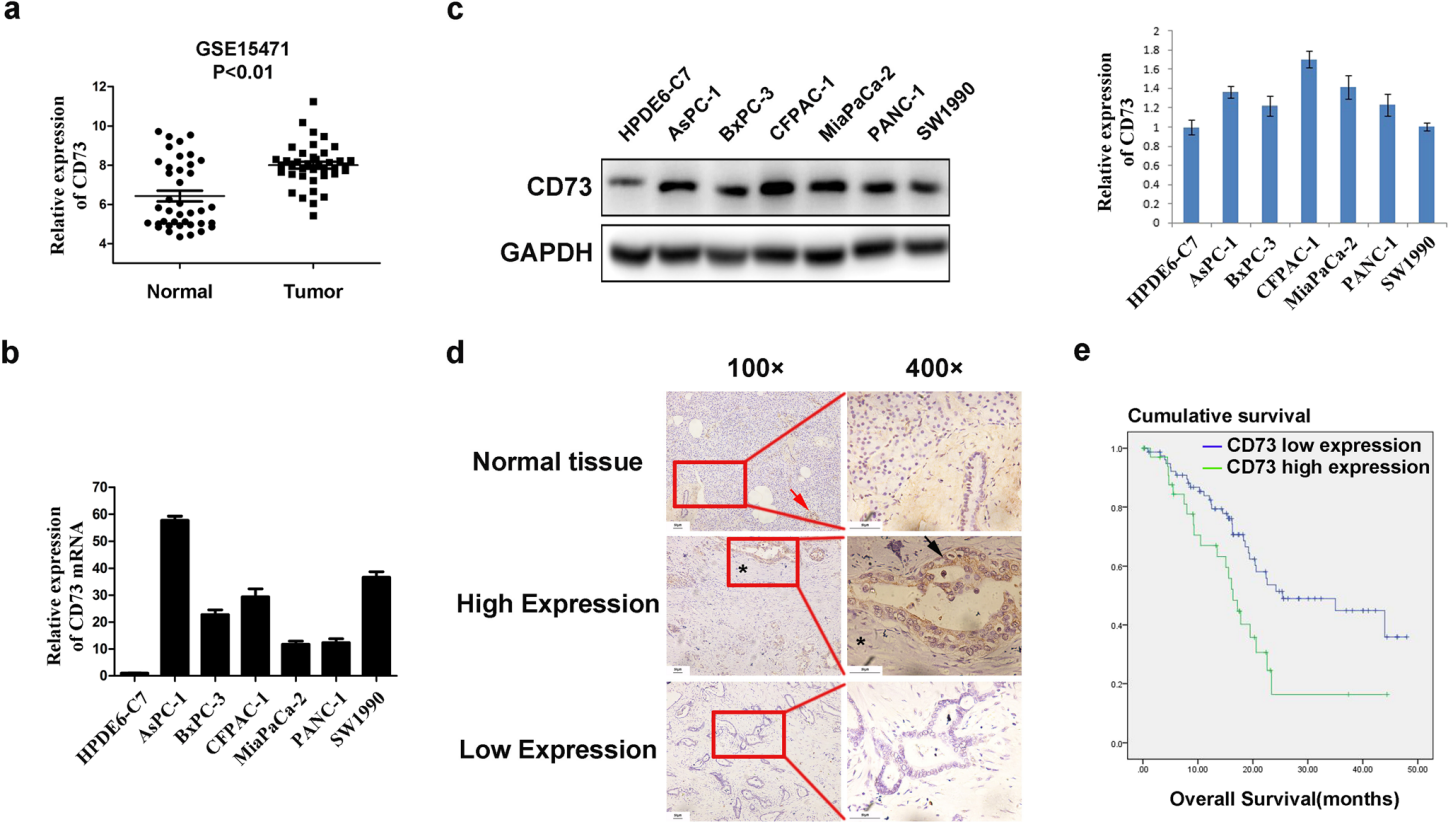
CD73 is overexpressed in PDAC tissues and cell lines. **a** Relative CD73 mRNA expression in PDAC tumors and adjacent normal pancreatic tissues by analyzing chip results in the database GSE15471. **b**, **c** The level of CD73 in PDAC cells was detected by qRT-PCR and western blot, respectively. **d** Immunohistochemistry was used to detect the expression of CD73 in pancreatic cancer tissues and normal pancreatic tissues. Black arrow indicates positive expression in tumor cells. Red arrow indicates positive expression in islet cells. Asterisk indicates negative expression in stroma. **e** Effect of the CD73 expression level on overall survival in 114 pancreatic cancer patients. Data are expressed as mean ± SEM (*n* = 3)

We first analyzed gene expression data from the GSE15471 database and found that the messenger RNA (mRNA) expression of CD73 was significantly higher in pancreatic cancer compared with normal tissues (*p* < 0.01) (Fig. 1a). qRT-PCR and western blot analysis confirmed high expression of CD73 in six pancreatic cancer cell lines compared with pancreatic ductal epithelial cells (HPDE6-C7) as controls (Fig. 1b, c). We also performed immunohistochemical analysis of CD73 in 114 cases of pancreatic cancer and normal pancreatic tissue specimens. The positive rate of CD73 expression in pancreatic cancer tissues was 30.7% (35/114), and CD73 staining was mainly located on the cell membrane and in the cytoplasm. In normal acini and ducts, CD73 was negatively expressed, while in islet cells, it showed positive expression (Fig. 1d).

We next classified patients into CD73 high and low expression groups and performed statistical analysis of clinical and pathological data. Significant differences of CD73 expression were observed in samples classified according to tumor size, T stage, and TNM stage (Table 1). After patient follow-up, we found that high levels of CD73 in tumor cells were associated with a significantly decreased overall survival (16.4 months ± 1.349 months vs. 25.400 months ± 7.965 months; *p* = 0.007) (Fig. 1e). In addition, the multivariable analysis also showed CD73 was an independent factor (HR = 3.480, *p* = 0.001, Table S2). Together, these findings suggest that CD73 plays critical roles in PDAC progression and may be a valuable biomarker for the prognosis.

**Table 1:** Relationship between CD73 expression and clinicopathological features

Parameters	*n* = 114	CD73 expression levels		*p* values
		Low	High	
Age				
< 60	67	43	24	0.157
≥ 60	47	36	11	
Gender				
Male	70	49	21	0.838
Female	44	30	14	
Pathological grade				
Poor	38	27	11	0.774
Middle and high	76	52	24	
Tumor size				
≤ 4 cm	89	72	17	< 0.001*
> 4 cm	25	7	18	
T stage				
T1–2	36	30	6	0.027*
T3–4	78	49	29	
Lymph node metastasis				
No	62	44	18	0.673
Yes	52	35	17	
Distant metastasis				
M0	99	70	29	0.402
M1	15	9	6	
TNM stage				
I–II A	54	43	11	0.023*
II B–IV	60	36	24	

*p < 0.05

### Knockdown of CD73 inhibits cell growth and cell cycle progression and promotes cell apoptosis

**Fig. 2:**
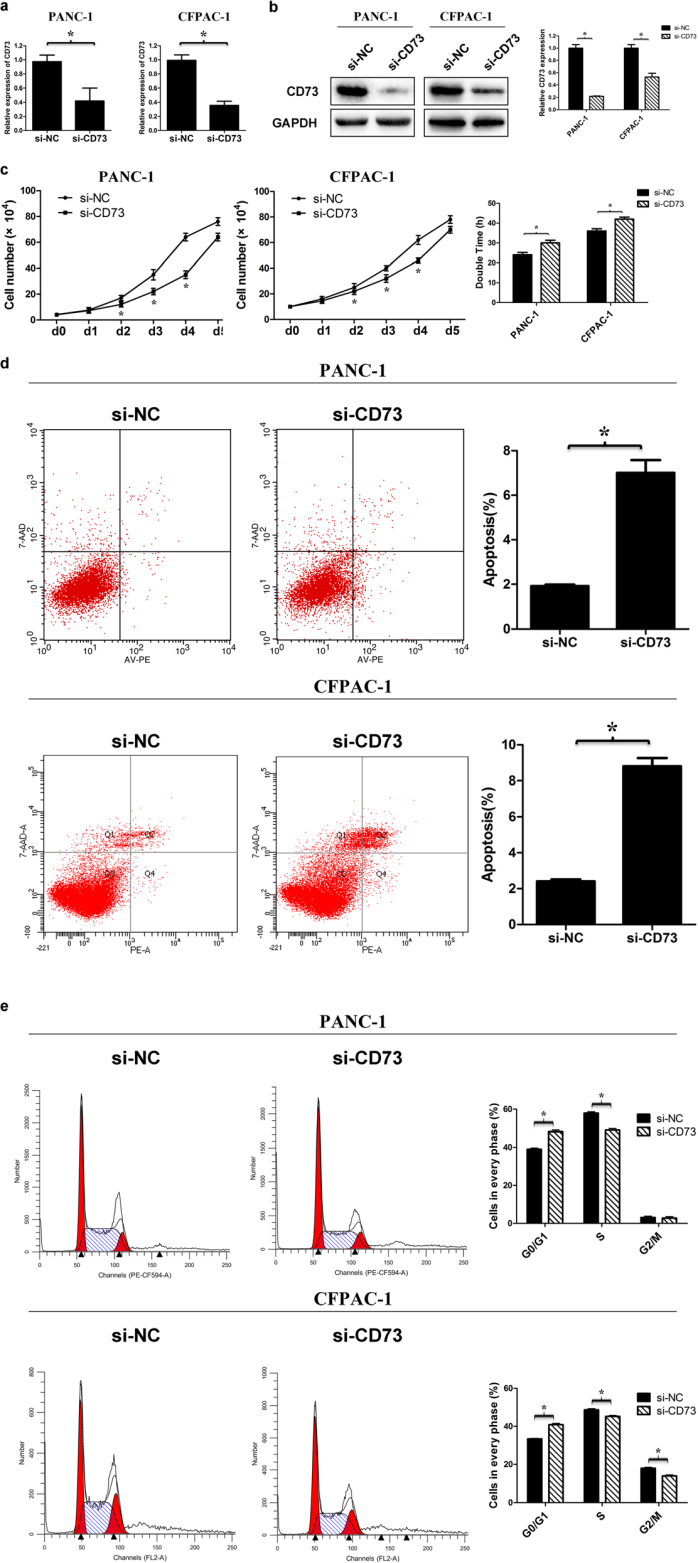
Knockdown of CD73 inhibits cell growth and cell cycle progression and promotes apoptosis of PDAC cells. **a**, **b** CD73 mRNA and protein levels in PANC-1 and CFPAC-1 cell lines transfected with CD73 siRNA or negative control. **c** Cells with CD73 knockdown showed reduced cell growth compared with the control cells. Doubling time of these cells was calculated at 48–96 h; the cells with CD73 knockdown showed a higher doubling time compared with the control cells. **d** Flow cytometric analysis showed apoptosis in CD73 knockdown cells was increased compared to the control. **e** Flow cytometry analysis indicated that the percentage of cells at G0/G1 phase in cell lines with CD73 knockdown was increased and the proportion at S phase was decreased. Data are expressed as mean ± SEM (*n* = 3). *p < 0.05

We knocked down CD73 by transfecting siRNA in PANC-1 and CFPAC-1 cell lines (Fig. 2a, b). The population doubling time was analyzed from experimental growth curves. As shown in Fig. 2c, the cell number was significantly lower and the doubling time value was increased in CD73 knockdown cells than in the control. Apoptosis was increased in CD73 knockdown cells compared with controls at 48 h post-transfection (Fig. 2d). Moreover, flow cytometry of PANC-1 cells with CD73 knockdown revealed that the percentage of cells at G0/G1 phase was increased and the proportion at S phase was decreased (Fig. 2e). Together, these data suggest that CD73 silencing inhibits cell proliferation in PDAC cells mainly via its effects on the cell cycle, indicating that CD73 may have important oncogenic roles in PDAC.

### CD73 knockdown induces G1 phase arrest via the AKT/ERK/cyclin D signaling pathway

**Fig. 3:**
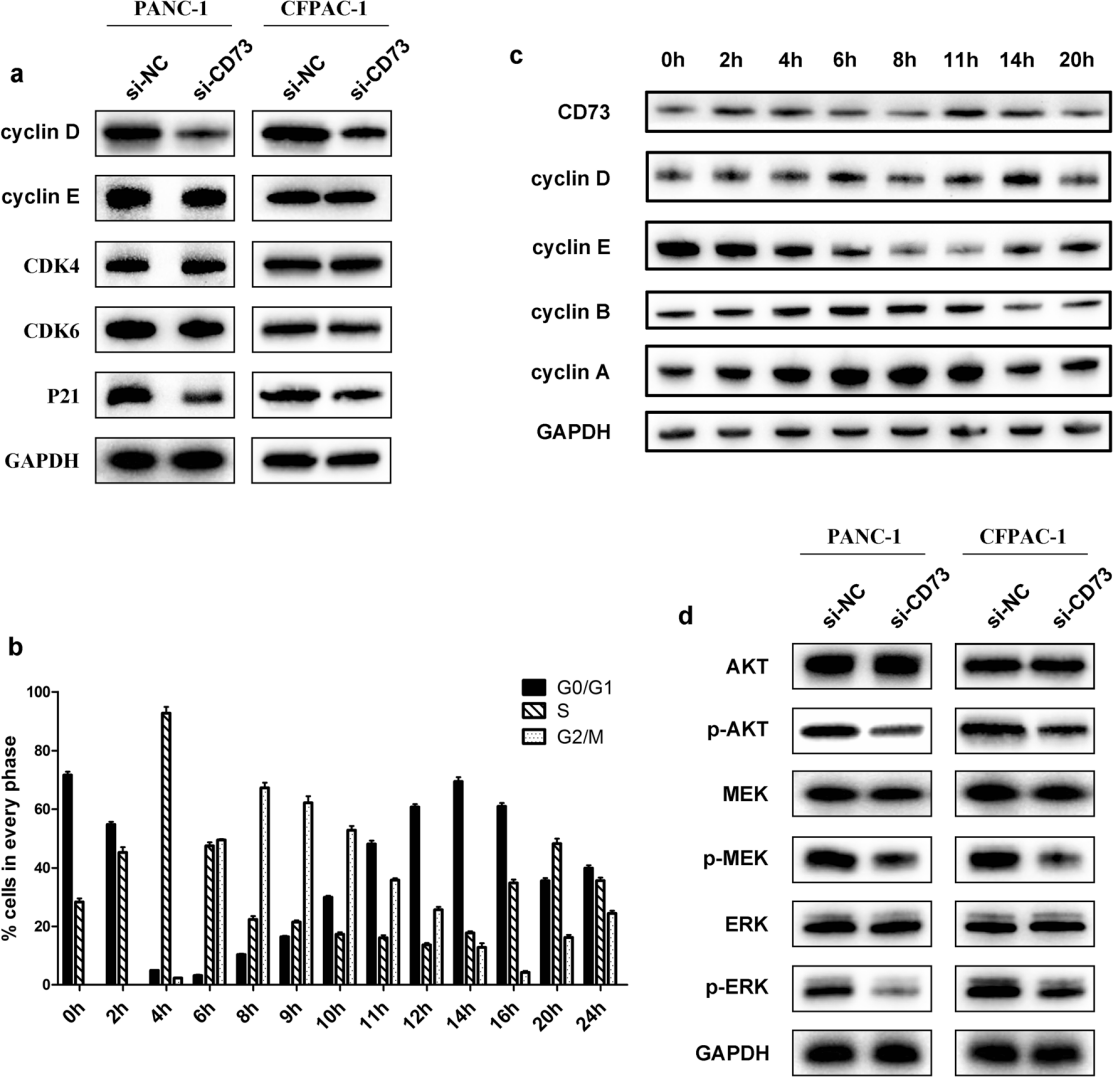
CD73 knockdown induces G1 arrest via AKT/ERK/cyclin D signaling pathway. **a** Western blotting assay to detect the expression of cyclins, proteins, CDKs, and CDK inhibitors. Expression of cyclin D was significantly reduced in CD73 knockdown cells. **b** The proportion of cells at the indicated times post-release from the double-thymidine block by flow cytometric analysis in PANC-1 cell lines. **c** Western blot analysis of CD73 and cyclin expression in PANC-1 cells after release from a double-thymidine block-induced cell cycle arrest. **d** The expression of proteins in the AKT and ERK signaling pathway was detected in CD73 knockdown or control cells. Data are expressed as mean ± SEM (*n* = 3). *p < 0.05

We next examined the expression levels of cell cycle regulatory proteins. Cyclin D was shown to be significantly reduced in CD73 knockdown cells, while the expression levels of cyclin E were not altered (Fig. 3a, Figure S1a). G1 phase-associated CDK4 and CDK6 levels were unchanged in CD73 knockdown cells, whereas the level of p21 was slightly decreased (Fig. 3a). To determine whether CD73 levels varied throughout the cell cycle, we examined CD73 protein levels in PANC-1 cells synchronized by a double-thymidine block and harvested at various times (Fig. 3b). The results showed that the expression of CD73 altered as the cell cycle progressed and peaked at 4 h and 11 h, which was slightly ahead of cyclin D (Fig. 3c, Figure S1b).

AKT and MAPK signaling pathways have been shown to play an important role in regulating the cell cycle and can inhibit cyclin D expression, leading to G1 phase arrest [21, 22]. Our data showed that p-AKT and p-ERK levels were significantly decreased in CD73 knockdown cells compared with control cells (Fig. 3d, Figure S1c). In contrast, no changes in the expression levels of p38 and JNK were observed (Figure S1d). It suggests that CD73 knockdown may inhibit AKT/ERK activation, which then decreases cyclin D expression to inhibit the G1/S transition in pancreatic cancer cells.

### TNFR2 is involved in CD73-induced AKT and ERK signaling pathway activation in PDAC

**Fig. 4:**
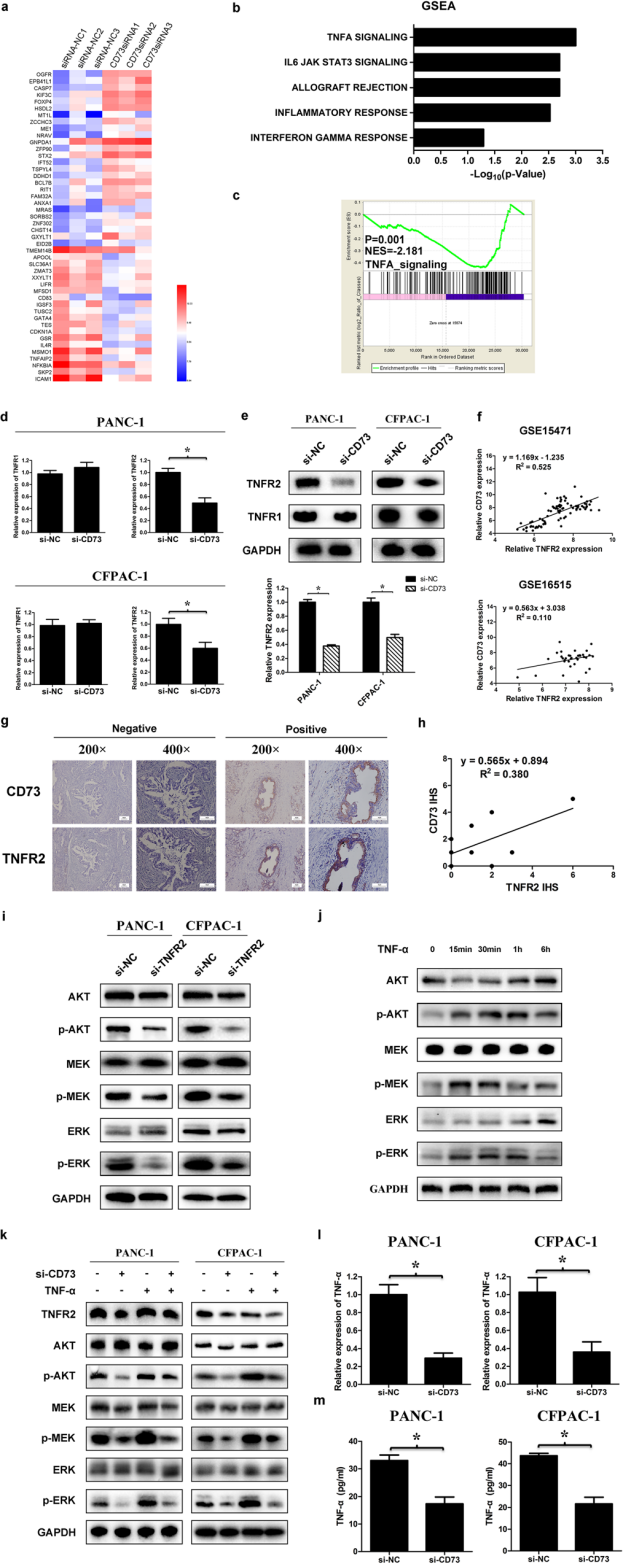
TNFR2 was involved in CD73-induced AKT and ERK signaling pathways activation in PDAC. **a** Heatmap representing relative expression of genes in CD73 knockdown cells. Results for separate knockdown experiments are shown (*n* = 3). **b** Significantly differentially expressed gene sets identified by GSEA, and the most significant are depicted here (*p* < 0.05). **c** The TNF-α signaling pathway genes were generally downregulated in CD73 knockdown cells. **d**, **e** mRNA and protein expression of TNFR1 and TNFR2 in PANC-1 and CFPAC-1 cell lines transfected with CD73 siRNA or siRNA negative control. **f** Positive correlation of CD73 and TNFR2 expression was observed by analyzing the GEO databases GSE16515 and GSE15471 (*r* = 0.567, *p* < 0.001; *r* = 0.725, *p* < 0.001). **g** Immunohistochemistry was used to detect the expression of CD73 and TNFR2 in pancreatic cancer tissues and normal pancreatic tissues. **h** Positive correlation between CD73 and TNFR2 (*n* = 10, *r* = 0.617, *p* = 0.058). **i** The expression of proteins in the AKT and ERK signaling pathway was detected in TNFR2 knockdown or control cells. **j** PANC-1 cells were treated with 20 ng/ml TNF-α. Protein in the AKT and ERK signaling pathway at different time points after treatment was analyzed by western blotting. **k** CD73 knockdown cells were treated with or without TNF-α (20 ng/ml) for 15 min. The expression levels of TNFR2, p-AKT, p-MEK, and p-ERK were analyzed by western blot analysis. **l** mRNA level of TNF-α in cell lines transfected with CD73 siRNA or siRNA negative control by PCR. **m** Expression of TNF-α in the supernatant by ELISA assay. Data are expressed as mean ± SEM (*n* = 3). *p < 0.05

We further investigated the downstream targets of CD73 using transcriptome sequencing of PANC-1 cell lines transfected with CD73 siRNA or negative controls. All raw data have been deposited in the GEO database (http://www.ncbi.nlm.nih.gov/geo/) under accession number GSE117012. Compared with the negative control group, a total of 506 genes were significantly changed in CD73 knockdown pancreatic cancer cells (*p* < 0.01) (Fig. 4a), including 244 upregulated genes and 262 downregulated genes. Additionally, five signaling pathway were identified as significantly altered along with aberrant CD73 expression by gene set enrichment analysis (GSEA) (*p* < 0.05) (Fig. 4b). Among these pathways, we noted that genes involved in the TNF-α signaling pathway were altered most significantly in CD73 knockdown PANC-1 cells (*p* < 0.05) (Fig. 4c).

TNF-α specifically binds to two cell surface receptors: TNFR1 and TNFR2. While TNFR1 is mainly responsible for apoptosis and cell death, TNFR2 does not have a death domain and therefore does not promote cell death [23–26]. Interestingly, we found that the expression of TNFR2 was significantly downregulated after CD73 knockdown, while TNFR1 was not changed (Fig. 4d, e). By further analyzing the GEO databases GSE16515 and GSE15471, which contained 52 and 72 cases of pancreatic cancer and normal pancreatic tissues, respectively, it showed the expression of CD73 was significantly associated with TNFR2 (*r* = 0.567, *p* < 0.001; *r* = 0.725, *p* < 0.001; Fig. 4f). The analysis was performed in the cancer and normal tissues, respectively, and similar results were also obtained (Figure S2a). Therefore, we further confirmed it in ten cases of pancreatic cancer tissues by IHC. By comparing the immunohistochemistry score (IHS) of CD73 and TNFR2, it was found that there was a positive correlation between them (*p* = 0.058, Fig. 4g, h).

Previous studies showed that activation of TNFR2 induces the AKT/ERK signaling pathway and promotes tumorigenesis [27], and we also confirmed that the AKT/ERK signaling pathway was inhibited after TNFR2 knockdown in pancreatic cancer (Fig. 4i, Figure S2b, c). We thus hypothesized that knockdown of CD73 may inhibit TNFR2 expression, thus leading to inhibition of AKT/ERK signaling pathway. We found that the AKT/ERK signaling pathway was significantly activated by TNF-α stimulation (Fig. 4j, Figure S2d). However, in cell lines with CD73 knockdown, the TNF-α-induced increase in the AKT/ERK pathway was inhibited (Fig. 4k, Figure S2e), indicating that TNFR2 was involved in CD73-induced AKT and ERK signaling pathway activation.

Additionally, a significant decrease in TNF-α gene expression was observed in CD73 knockdown cells by PCR (Fig. 4l) and in the culture supernatant by ELISA (Fig. 4m), suggesting further inhibition of TNFR2 pathway activation.

### CD73 is a direct target of miR-30a-5p

**Fig. 5:**
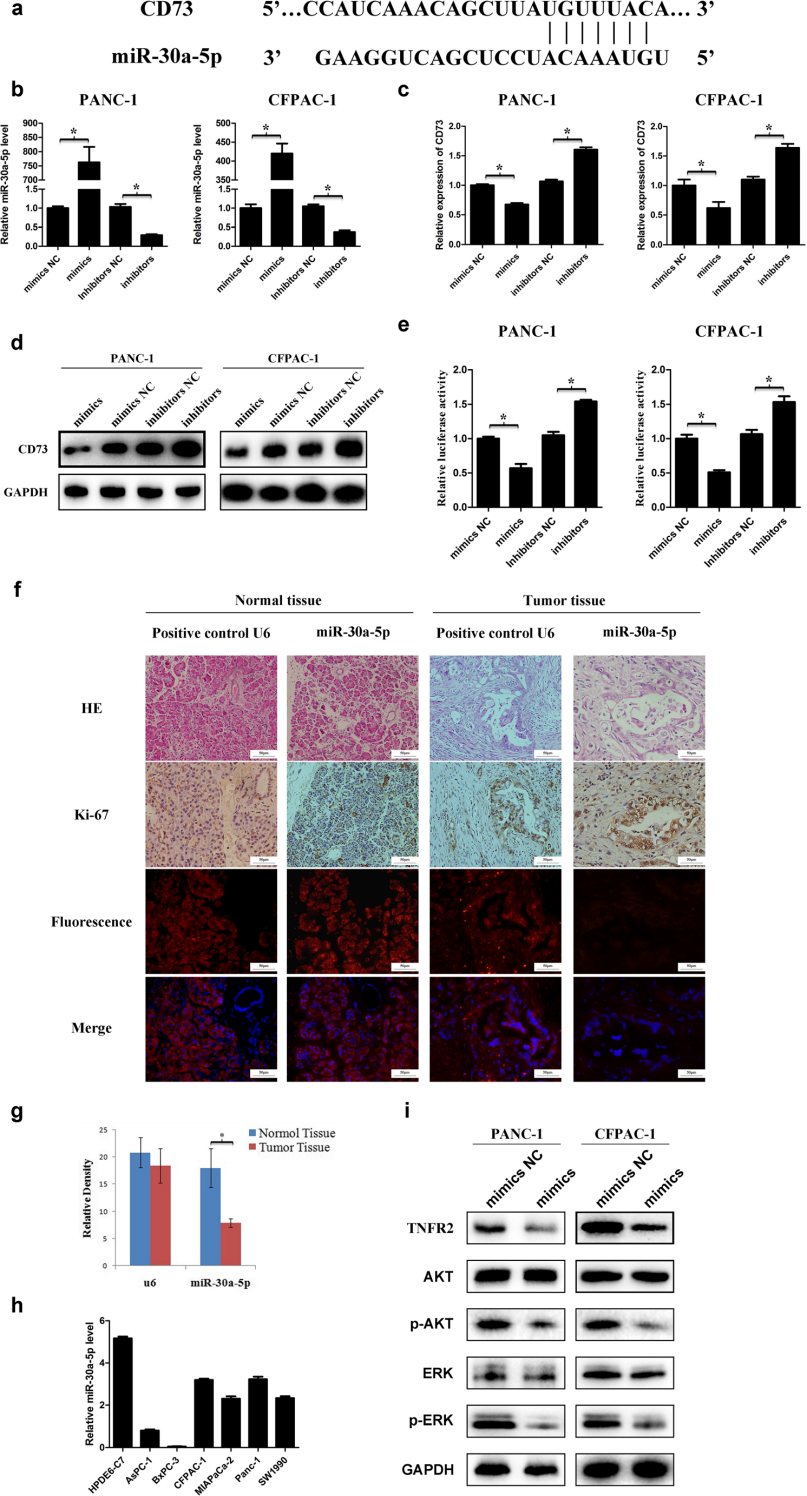
CD73 is a direct target for miR-30a-5p. **a** The 3′-UTR of CD73 contains a complementary matching region of miR-30a-5p through predictive analysis by bioinformatics websites. **b** Transfection of miR-30a-5p mimics or inhibitors was performed, and the efficiency of transfection was examined by qPCR. **c**, **d** mRNA and protein expression levels of CD73 in cell lines transfected with miR-30a-5p mimics or inhibitors. **e** Luciferase activity of the construct containing the CD73 3′-UTR reporter gene in 293 T cells co-transfected with the miR-30a-5p mimics or inhibitors. **f** The expression level of miR-30a-5p in pancreatic cancer tissue was detected by FISH. Ki-67 was used as cancer cell marker. **g** miR-30a-5p expression in tumor tissue was lower than that in normal tissue (*n* = 10, *p* < 0.05). **h** The expression levels of miR-30a-5p in pancreatic cell lines with the normal pancreatic duct epithelial cell line HPDE6-C7 as control. **i** The expression levels of TNFR2, p-AKT, and p-ERK were analyzed by western blotting in cell lines treated with miR-30a-5p mimics or negative control. Data are expressed as mean ± SEM (*n* = 3). *p < 0.05

To identify the miRNA that can bind to CD73 mRNA, we used TargetScan, miRanda, and miRDB for predictive analysis. The 3′-UTR of CD73 mRNA was shown to contain a complementary region to miR-30a-5p (Fig. 5a). To examine whether CD73 is regulated by miR-30a-5p, we overexpressed miR-30a-5p in cells and confirmed the efficiency of transfection by qPCR (Fig. 5b). Compared with control cells, miR-30a-5p-overexpressing cells had significantly decreased CD73 mRNA and protein levels; in contrast, inhibition of miR-30a-5p expression significantly increased CD73 expression (Fig. 5c, d, Figure S3a). To further assess whether CD73 is a direct target of miR-30a-5p, we performed luciferase reporter assays. miR-30a-5p overexpression significantly decreased the activity of a luciferase reporter harboring the 3′-UTR of CD73, confirming that miR-30a-5p directly targets CD73 mRNA (Fig. 5e).

Previous studies showed that miR-30a-5p is frequently downregulated in human cancers, and decreased miR-30a-5p expression was also found in PDAC tissues (Fig. 5f, g) and cell lines (Fig. 5h). Furthermore, in line with the results in CD73 knockdown cells, we found that TNFR2, p-AKT, and p-ERK expression was significantly lower in miR-30a-5p-overexpressing cells compared with controls (Fig. 5i, Figure S3b).

### Overexpression of miR-30a-5p inhibits tumor growth by targeting CD73 and increases the sensitivity of pancreatic cancer to gemcitabine

**Fig. 6:**
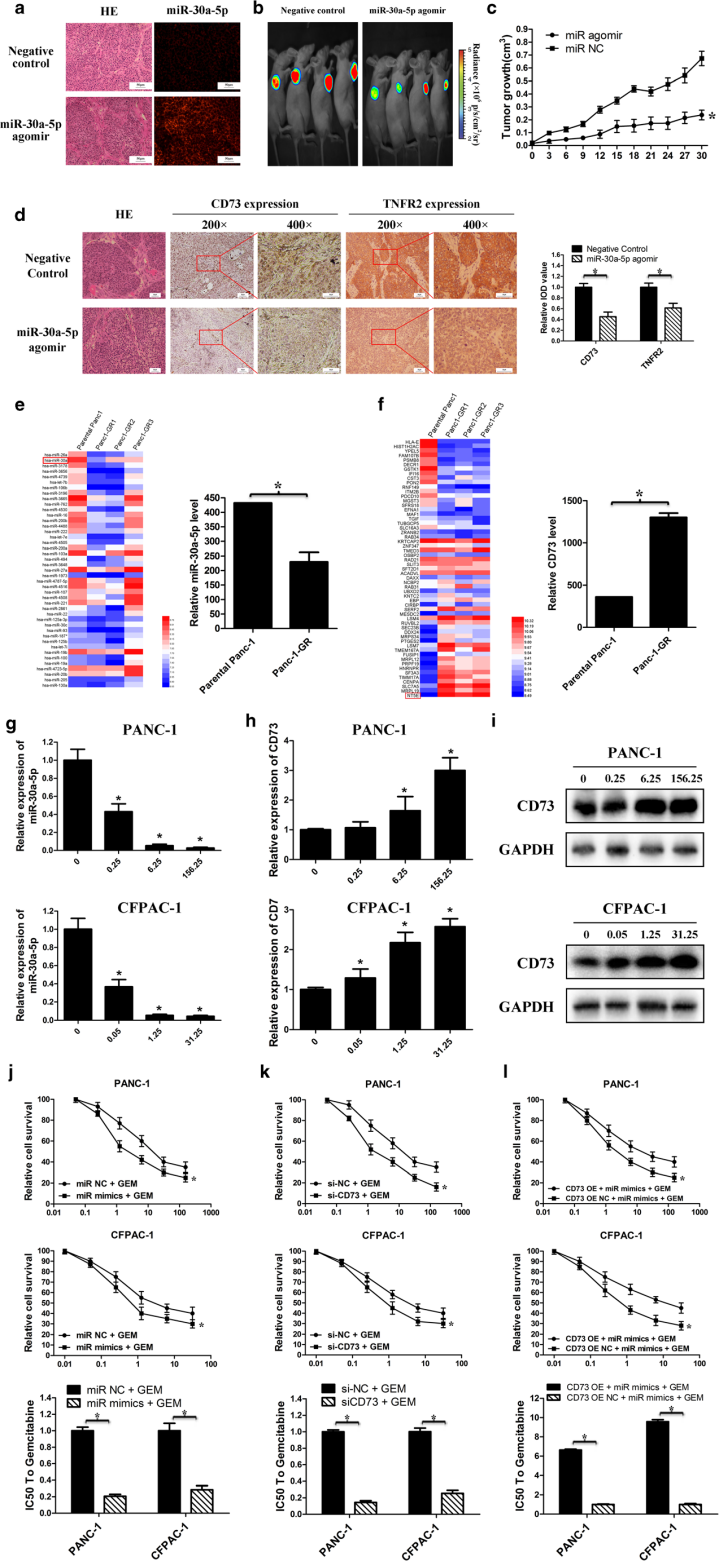
Overexpression of miR-30a-5p inhibits tumor growth by targeting CD73 and increases the sensitivity of pancreatic cancer to gemcitabine. **a** The miR-30a-5p was confirmed significantly increased in the subcutaneous tumors after the intratumoral injection of miR-30a-5p agomir by FISH. **b** miR-30a-5p agomir or control oligo mixture was injected into the xenografts in a multi-site injection manner every 3 days for 2 weeks, and tumor growth was measured in mice by bioluminescence imaging 30 days post-implant (*n* = 4 in each group). **c** Tumor growth curves in mice at the indicated days. **d** The expression level of CD73 and TNFR2 in the tumor tissues from miR-30a-5p agomir or negative control–treated nude mice was detected by IHC. **e**, **f** Analysis of the changes in miRNA and mRNA expression levels in gemcitabine-resistant pancreatic cancer cell line PANC-1 based on the GEO databases GSE80616 and GSE80617. **g** PANC-1 and CFPAC-1 were stimulated with different concentrations of gemcitabine (nM) for 48 h. The expression of miR-30a-5p was detected by PCR. **h**, **i** PANC-1 and CFPAC-1 were stimulated with different concentrations of gemcitabine (nM) for 48 h. The expression of CD73 was detected by PCR and western blot. **j** The IC_50_ value to gemcitabine decreased in miR-30a-5p-overexpressed cells compared to negative control. **k** The IC_50_ value to gemcitabine decreased in CD73 knockdown cells compared to negative control. **l** CD73 overexpression inhibited the effect of miR-30a-5p on promoting the cell-killing effect of gemcitabine. Data are expressed as mean ± SEM (*n* = 3). *p < 0.05

Considering that PDAC cells showed both low miR-30a-5p and high CD73 expression levels and based on the requirement for CD73 in PDAC cell proliferation (Fig. 2), we hypothesized that miR-30a-5p may inhibit PDAC cell proliferation via CD73. We generated xenografted nude mice, in which significantly increased miR-30a-5p expression was confirmed in subcutaneous tumors after the intratumoral injection of an miR-30a-5p agomir (Fig. 6a). We found that tumors in the miR-30a-5p overexpression group grew more slowly compared with those in the negative control group (Fig. 6b, c). Additionally, immunohistochemistry showed that CD73 and TNFR2 were significantly decreased in tumor tissues from miR-30a-5p-overexpressing nude mice (Fig. 6d). These findings indicate that miR-30a-5p could inhibit tumor growth and negatively regulate CD73 expression.

Analysis of the changes in miRNA and mRNA expression between the gemcitabine-resistant pancreatic cancer cell line PANC-1 and the parental pancreatic cancer cell line based on GSE80616 and GSE80617 datasets in the GEO database revealed that both miR-30a-5p and CD73 had the most significant changes. In drug-resistant pancreatic cancer cells, the expression level of miR-30a-5p was even lower (*p* < 0.05) (Fig. 6e) and the expression level of CD73 was higher (*p* < 0.05) (Fig. 6f). Based on our findings of the interaction between miR-30a-5p and CD73, we next examined whether miR-30a-5p and CD73 affected the antitumor activity of gemcitabine in pancreatic cancer cells. In the study, PANC-1 and CFPAC-1 were stimulated with different concentrations of gemcitabine for 48 h. The expression of miR-30a-5p decreased significantly and correlated with the concentration (Fig. 6g). CD73, as a target of miR-30a-5p, also showed a concentration-dependent increase after stimulation with gemcitabine (Fig. 6h, i, Figure S4a). In pancreatic cancer cells overexpressing miR-30a-5p or knockdown of CD73, it showed an increased cytotoxic effect of gemcitabine compared with control cells (*p* < 0.05) (Fig. 6j, k). Furthermore, simultaneous miR-30a-5p and CD73 overexpression revealed that CD73 overexpression inhibited the effect of miR-30a-5p on enhancing the cytotoxicity of gemcitabine to some extent (Fig. 6l, Figure S4b). Therefore, the miR-30a-5p/CD73 axis may be involved in the development of gemcitabine resistance in pancreatic cancer.

## Discussion

**Fig. 7:**
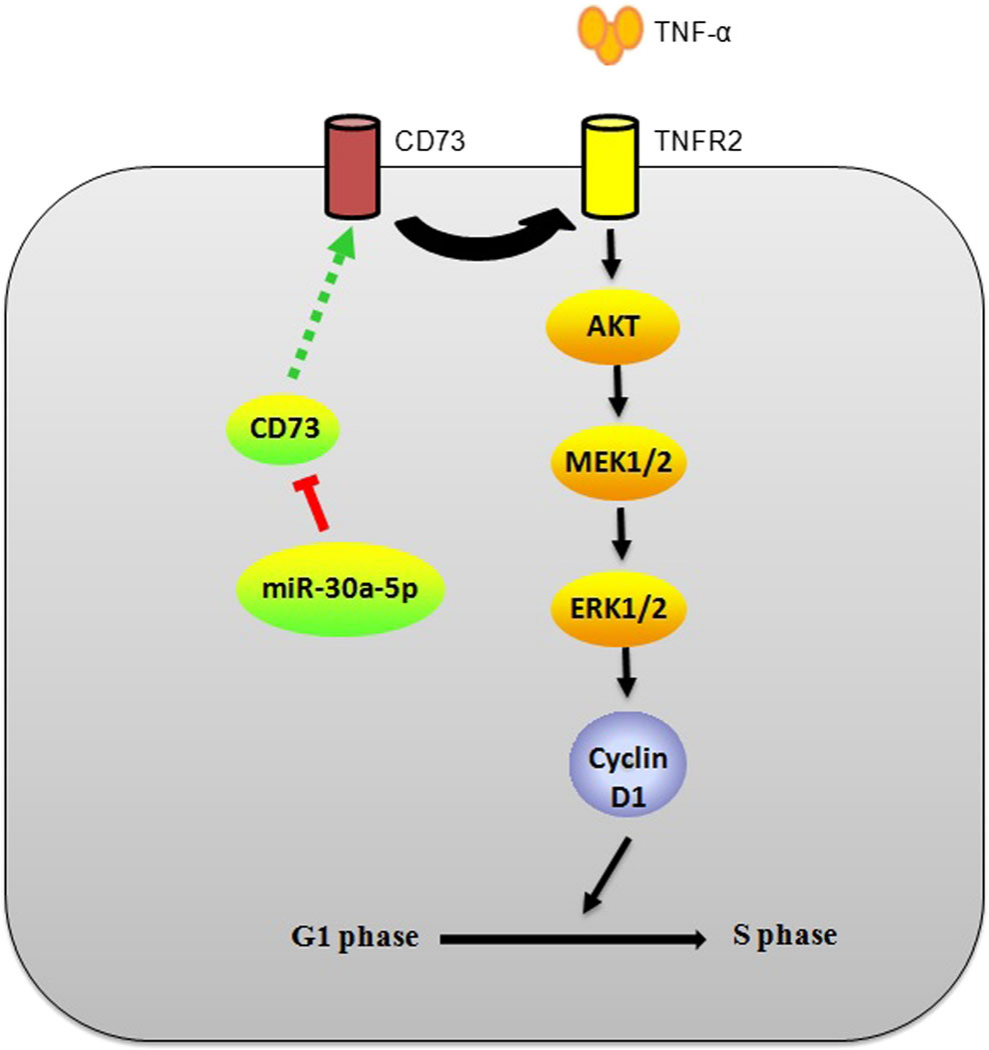
Proposed model for the interaction between miR-30a-5p, CD73, and TNFR2 and inhibition of the AKT/ERK pathway to induce G0/G1 phase arrest. Knockdown of CD73 inhibits TNFR2 expression, thus leading to inhibition of AKT/ERK signaling pathway. miR-30a-5p directly targets the CD73 3′-UTR and negatively regulates CD73 and downstream pathways

The overexpression of CD73 has been found to promote cancer cell proliferation in vitro. Previous studies have demonstrated that CD73 promotes the growth of cancer cells depending on its enzymatic activity, that is, the production of adenosine [28]. However, conflicting studies showed that adenosine can also inhibit cell growth and induce apoptosis [29]. The role of CD73 in promoting the proliferation of cancer cells may be achieved by other molecules independent of adenosine. So far, however, little is known about the molecular or signaling pathways involved in the promotion of tumor growth by CD73. Here, we found that the suppression of CD73 by siRNA induced G1 cell cycle arrest through AKT and ERK signaling pathways, and the interaction between miR-30a-5p, CD73, and TNFR2 may provide new insights into the regulatory network of CD73 (Fig. 7).

In recent years, TNFR2 has attracted increasing attention because of its important role in promoting proliferation, migration, and angiogenesis in various cancers [30–32]. Torrey et al. [33] and Vanamee and Faustman [34] found that targeting TNFR2 with antagonistic antibodies significantly inhibited the proliferation of ovarian cancer cells, and the follow-up study confirmed the therapeutic effects of TNFR2 antibodies on other tumors [35]. Interestingly, in this study, a significant decrease in TNFR2 expression was observed in CD73 knockdown cells. This is of particular importance in recognizing the regulatory network of CD73 in promoting tumor progression. However, the specific mechanism by which CD73 regulates TNFR2 is still unclear. So far, EGFR is a well-studied molecule targeted by CD73 [36]. It was reported by Zhi et al. [37] that CD73 regulates the activity of some associated transcription factors (TFs) in MB-MDA-231 cells, among which PPARγ may mediate EGFR expression. Besides, CD73 as GPI-linked molecules can be expressed on the cell surface in clusters closely associated with other molecules, such as tyrosine kinases of the Src family, which was located upstream from EGFR activation [38]. However, the transcription factor of TNFR2 and its regulation mechanism are still rarely studied. Therefore, the verification of the correlation between CD73 and TNFR2 in large samples and the exploration of the potential regulatory mechanisms are required.

miRNAs are a group of endogenous non-coding RNAs, which has attracted much attention [39–41]. In the present study, we found that expression of miR-30a-5p was significantly reduced in pancreatic cancer, and that forced expression of miR-30a-5p inhibited tumor growth and increased the sensitivity of pancreatic cancer cells to gemcitabine. Zhu et al. reported that miR-30a plays an important role in non-small-cell lung cancer via its effects on CD73 gene expression [42]. Consistent with previous findings, we found that the miR-30a-5p/CD73 axis is one of the mechanisms that regulate the development of tumor progression in pancreatic cancer, suggesting that interfering with miR-30a-5p/CD73 functions might be a therapeutic strategy for pancreatic cancer.

In conclusion, we found that CD73 is overexpressed in PDAC and that its expression correlated with poor patient prognosis. The mechanistic interaction between miR-30a-5p, CD73, and TNFR2 may provide new insights into therapeutic strategies for PDAC.

## Electronic supplementary material

ESM 1 (DOCX 698 kb)

Supplementary Table 1 (DOCX 13 kb)

Supplementary Table 2 (DOC 59 kb)
