# Supplementary material for: The distinct role of CD73 in the progression of pancreatic cancer
Source: J Mol Med (Berl). 2019 Mar 29;97(6):803–15. doi: 10.1007/s00109-018-01742-0 (PMC6525710; doi:10.1007/s00109-018-01742-0)
Supplement: Supplementary file 1 — (DOCX 698 kb) [file 109_2018_1742_MOESM1_ESM.docx]

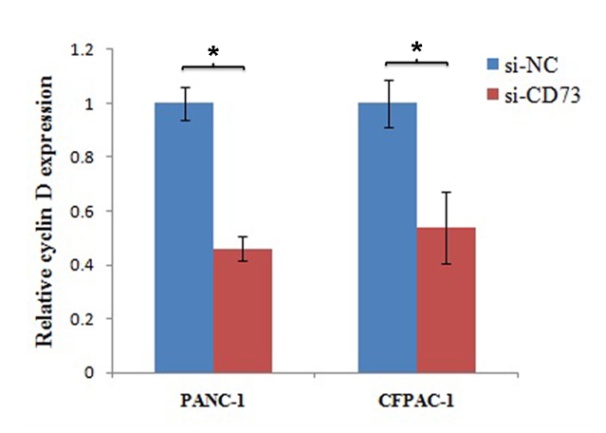

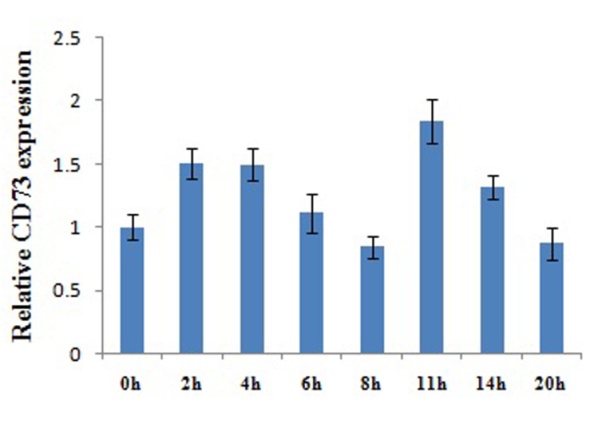


**c**

**a**

**b**


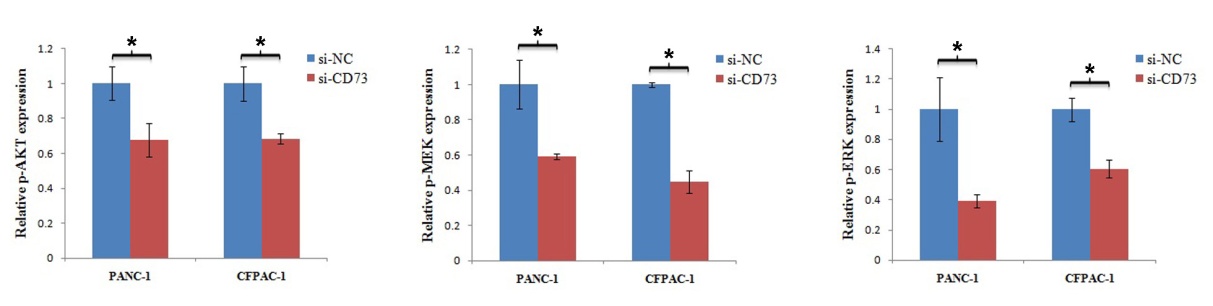


**d**


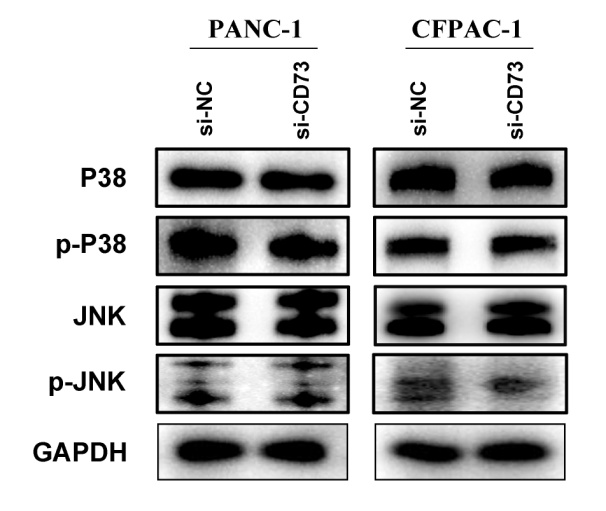


Supplementary Figure 1. **a**: The densitometry result of western blotting assay to detect the expression of cyclin D in CD73 knockdown cells. **b**: The densitometry result of western blotting assay to detect the expression of CD73 in PANC-1 cells after release from a double-thymidine block-induced cell cycle arrest. **c**: The densitometry result of western blotting assay to detect the proteins in the AKT and ERK signaling pathway in CD73 knockdown or control cells. **d**: The expression of proteins in the JNK and P38 signaling pathway was detected in CD73 knockdown or control cells. Data are expressed as mean ± SEM (n = 3). *indicated p<0.05.

**b**

**a**


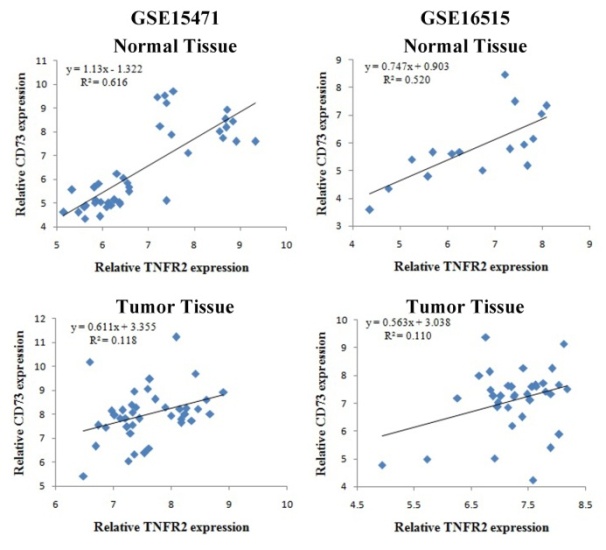

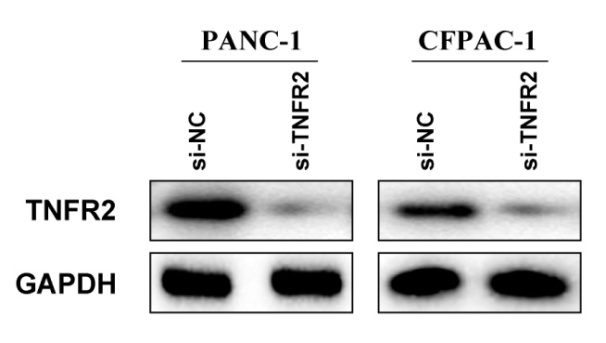


**c**


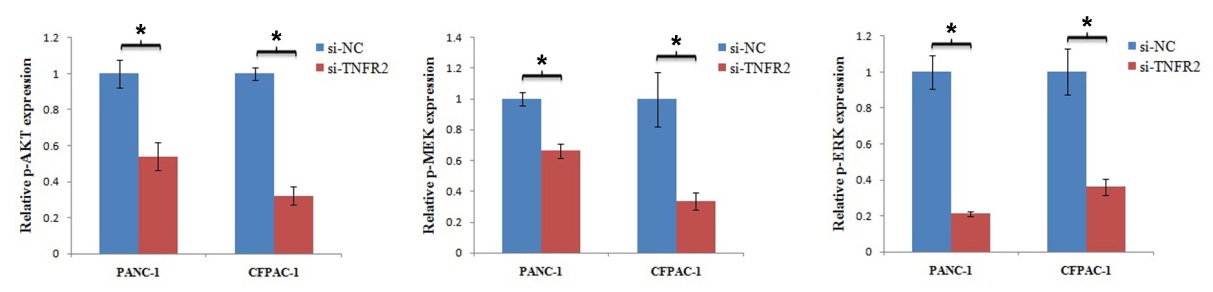


**d**


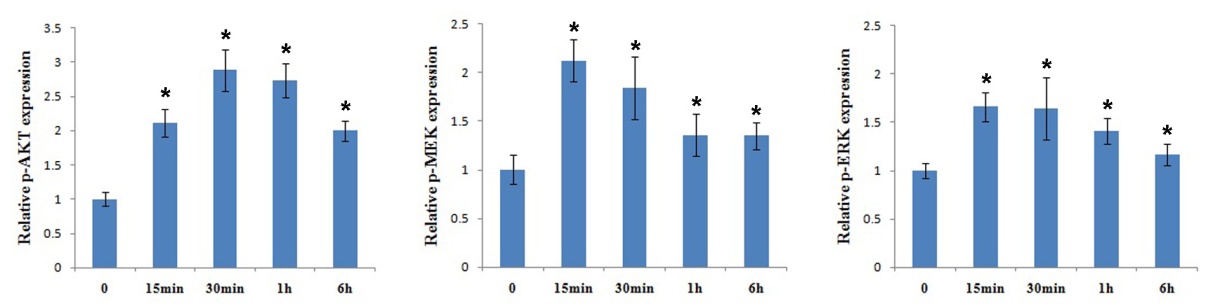


**e**


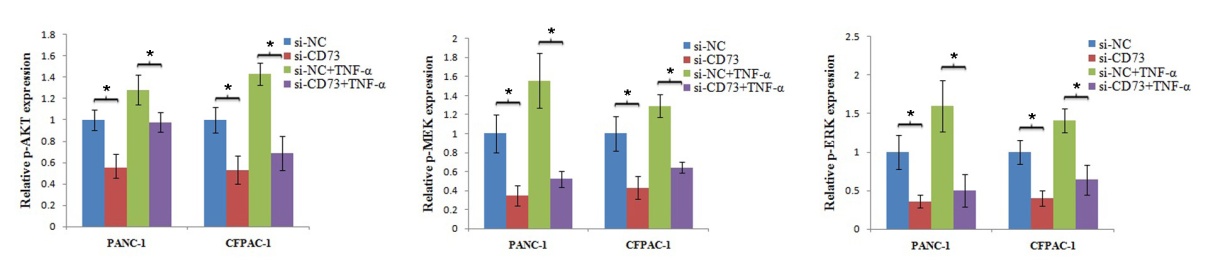


Supplementary Figure 2. **a**: Positive correlation of CD73 and TNFR2 expression was observed in the cancer and normal tissue respectively by analyzing the GEO database GSE16515 and GSE15471. **b**: TNFR2 protein levels in PANC-1 and CFPAC-1 cell lines transfected with TNFR2 siRNA or siRNA negative control. **c**: The densitometry result of western blotting assay to detect the proteins in the AKT and ERK signaling pathway in TNFR2 knockdown or control cells. **d**: The densitometry result of western blotting assay to detect the proteins in the AKT and ERK signaling pathway at different time points after treatment of TNF-α. **e**: The densitometry result of western blotting assay to detect the expression levels of TNFR2, p-AKT, p-MEK and p-ERK in CD73 knockdown cells treated with or without TNF-α. Data are expressed as mean ± SEM (n = 3). *indicated p<0.05.


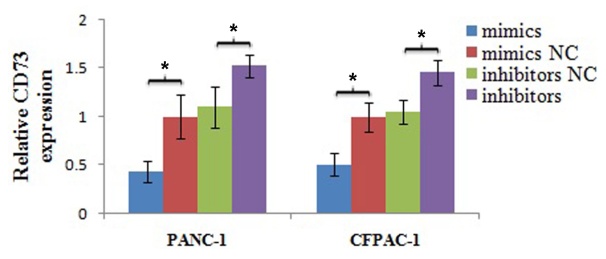


**b**

**a**


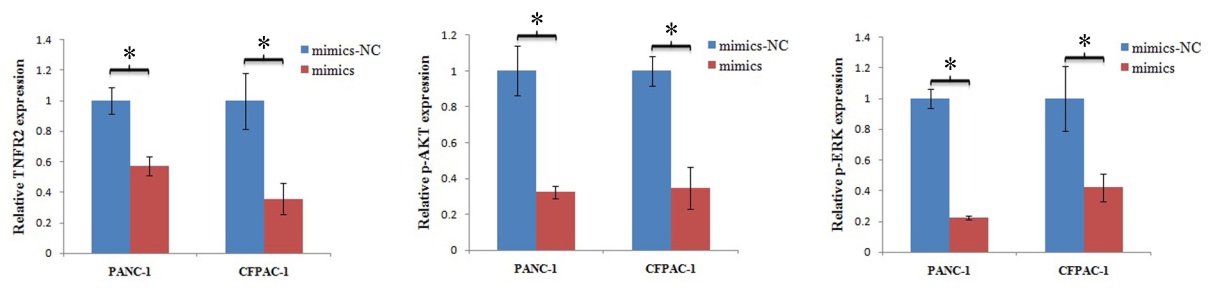


Supplementary Figure 3. **a**: The densitometry result of western blotting assay to detect the expression of CD73 in cell lines transfected with miR-30a-5p mimics or inhibitors. **b**: The densitometry result of western blotting assay to detect the expression levels of TNFR2, p-AKT and p-ERK in cell lines treated with miR-30a-5p mimics or negative control. Data are expressed as mean ± SEM (n = 3). *indicated p<0.05.


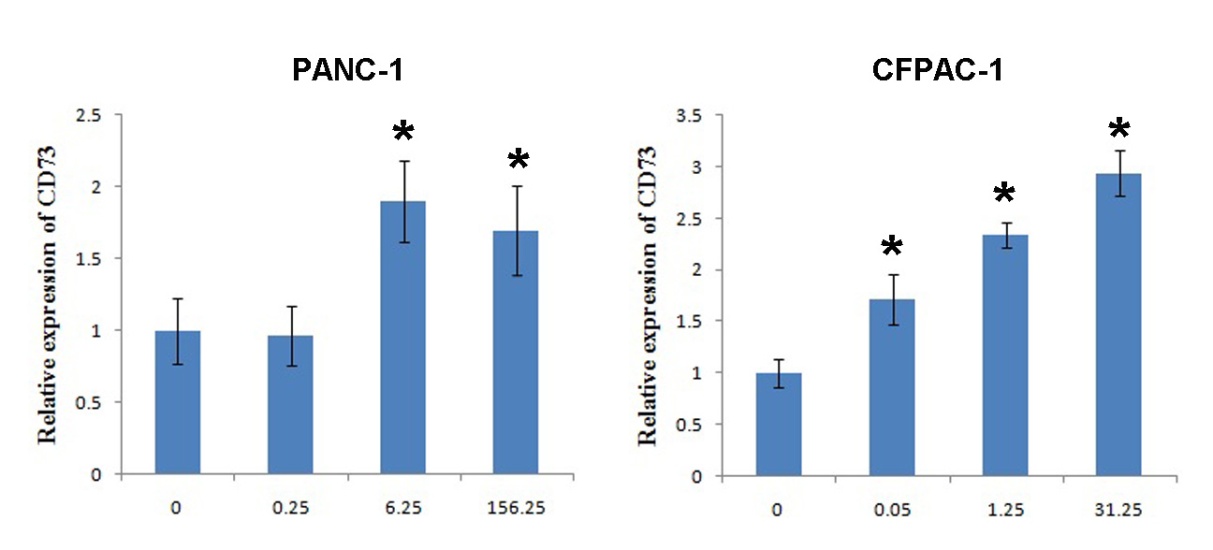


**a**


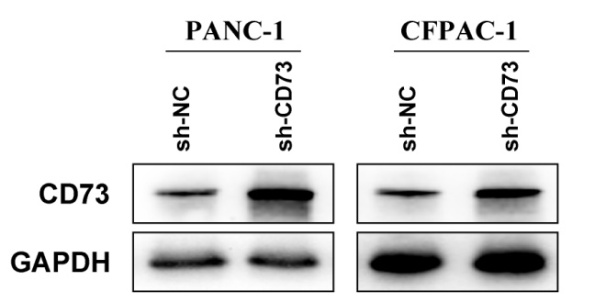


**b**

Supplementary Figure 4. **a**: The densitometry result of western blotting assay to detect the expression of CD73 in PANC-1 and CFPAC-1 stimulated with different concentrations of gemcitabine (nM) for 48h. **b**: CD73 protein levels in PANC-1 and CFPAC-1 cell lines transfected with recombinant plasmids over-expressing CD73 or negative control. Data are expressed as mean ± SEM (n = 3). *indicated p<0.05.
