# Supplementary material for: The distinct role of CD73 in the progression of pancreatic cancer
Source: J Mol Med (Berl). 2019 Mar 29;97(6):803–15. doi: 10.1007/s00109-018-01742-0 (PMC6525710; doi:10.1007/s00109-018-01742-0)
Supplement: Supplementary file 2 — (DOCX 13 kb) [file 109_2018_1742_MOESM2_ESM.docx]

**Supplementary Table 1: The list of the primary antibody.**

| primary antibody | Information |
| --- | --- |
| CD73 | 1:1000, sc-32299, Santa Cruz |
| cyclin D1 | 1:1000, MA1-39546, Thermo |
| cyclin E1 | 1:1000, ab133266, Abcam |
| cyclin A2 | 1:1000, ab181591, Abcam |
| cyclin B1 | 1:1000, ab181593, Abcam |
| CDK4 | 1:1000, SAB1403657, Sigma |
| CDK6 | 1:1000, 13331, Cell Signaling Technology |
| P21 | 1:1000, 2947, Cell Signaling Technology |
| AKT | 1:1000, ab192623, Abcam |
| p-AKT | 1:1000, ab192623, Abcam |
| MEK | 1:1000, ab178876, Abcam |
| p-MEK | 1:1000, 9154, Cell Signaling Technology |
| ERK | 1:1000, 4695, Cell Signaling Technology |
| p-ERK | 1:1000, 4370, Cell Signaling Technology |
| P38 | 1:1000, ab170099, Abcam |
| p-P38 | 1:1000, 9211, Cell Signaling Technology |
| JNK | 1:1000, ab179461, Abcam |
| p-JNK | 1:1000, 9255, Cell Signaling Technology |
| TNFR1 | 1:1000, ab68160, Abcam |
| TNFR2 | 1:1000, ab109322, Abcam |
| GAPDH | 1:5000, ab181602, Abcam |
