# Supplementary material for: The distinct role of CD73 in the progression of pancreatic cancer
Source: J Mol Med (Berl). 2019 Mar 29;97(6):803–15. doi: 10.1007/s00109-018-01742-0 (PMC6525710; doi:10.1007/s00109-018-01742-0)
Supplement: Supplementary file 3 — (DOC 59 kb) [file 109_2018_1742_MOESM3_ESM.doc]

Supplementary Table 2: Statistical analysis of clinicopathological variables associated with overall survival in patients with PDAC using the multivariate Cox proportional hazards model.
Characteristic	HR	95%CI	P value	
Age	1.002	0.973-1.032	0.892	
Gender	1.213	0.609-2.418	0.583	
Pathological grade	1.522	1.077-2.150	0.017*	
Tumor size	0.971	0.811-1.163	0.749	
T stage	0.606	0.268-1.373	0.230	
Lymph node metastasis	7.320	1.538-34.841	0.012*	
TNM stage	1.551	0.430-5.586	0.033*	
CD73	3.480	1.645-7.363	0.001*	
* p < 0.05
